# 1.J. Round table: Research Methods for Public Health Oriented Health Services Research – what works where and for what?

**DOI:** 10.1093/eurpub/ckac129.033

**Published:** 2022-10-25

**Authors:** Organised by: EUPHA-HSR, EUPHA-HIA, EUPHA-HTA, EUPHA-EPI, EUPHA-ECO, EUPHA-PHPP, German Public Health Association Section Research, German Network Health Service Research WG IHSR, Charité Institute for General Practice WG HPSRI, Chair persons: Lorena Dini (EUPHA-HSR), Johan Hansen (EUPHA-HSR)

## Abstract

This workshop will present the different methodological approaches relevant to the field of public health. This includes approaches of health systems and health policy research, epidemiology, health technology assessment, health impact assessment, health systems performance and economic evaluations. Presentations will be hold by presidents and vice-presidents of the EUPHA Sections in the form of a panel, presenting the overview of each methodology and providing case examples. All organizers might invite special contributions from their members and students to share innovations of case examples from their counties. During the first half of the workshop the EUPHA Sections will provide input on traditional and innovative methods the following topics:

1. Health Policy and Systems Research (HPSR) applied to Primary Care.

2. The use of traditional and innovative epidemiological study designs for public health relevant inferences.

3. Overview of methods used for the quantification of health impacts, both following a risk assessment approach or an epidemiological approach.

4. Methodologies and techniques applied in Health Technology Assessment (HTA) and how their contribute to decision-making and evidence-informed policymaking.

5. Health Systems Performance Assessment (HSPA) and Economic evaluations.

6. Research methods from the public health policy and politics perspective.

In the second half of the workshop we will discuss and share examples on innovation in research methods and seek to find an answer to the question: what is being done in the countries? Input from DGPH-FO, DNVF-IHRS, Charité WG HPSRI and from all participants will be welcome. Target audience: young and old professionals interested in updating their methods skills and sharing their own experiences on innovation in methods for a stronger public health research.

**Key messages:**

• There are no better or worse research methods; they are just a tool, that when applied properly support evidence and action for a stronger public health.

• As knowledge on research methods evolve, sharing knowledge amongst different fields is key to contribute to a stronger health system, better health policy decisions and a better health for all.

**Speakers/Panellists:**

**Lorena Dini**

Charité Univesitätsmedizin Berlin, Berlin, Germany

**Piedad Martin-Olmedo**

Escuela Andaluza de Salud Pública, Granada, Spain

**Stefania Boccia**

Italian Society of Hygiene, Preventive Medicine and Public Health, Rome, Italy

**Chiara de Waure**

University of Perugia, Perugia, Italy

**João Vasco Santos**

Faculty of Medicine, University of Porto, CINTESIS, Porto, Portugal

